# Supplementary material for: Metabolic Insights Into Microbially Induced Calcite Formation by Bacillaceae for Application in Bio‐Based Construction Materials
Source: Environ Microbiol. 2025 Apr 2;27(4):e70093. doi: 10.1111/1462-2920.70093 (PMC11965779; doi:10.1111/1462-2920.70093)
Supplement: Supplementary file 1 — Figure S1. Monod kinetic visualising the acetate usage. Figure S2. Structure prediction of the Excalibur Domain Protein using AlphaFold3. [file EMI-27-e70093-s001.pdf]

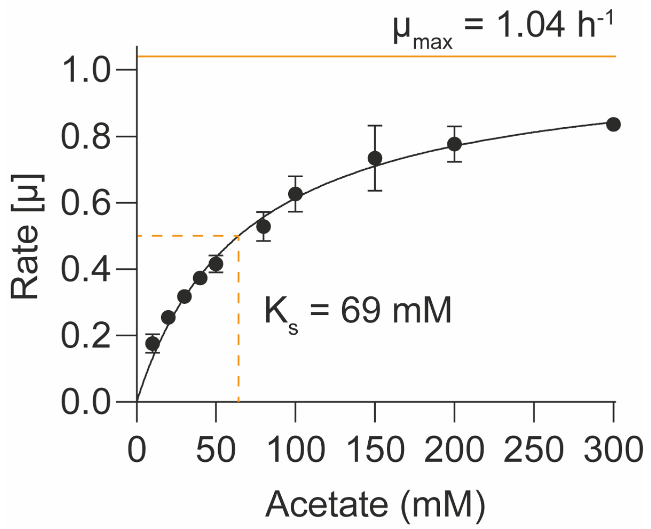

Figure S1. Monod kinetic visualizing the acetate usage. The growth rate (μ) is plotted as a function of acetate concentration (mM). The curve represents the Monod model fit, characterized by a maximum growth rate (μ_{max}) of 1.04 h^{-1} and a half-saturation constant (K_s) of 69 mM. Data are shown as mean and standard deviation of of the mean for three biological replicates.

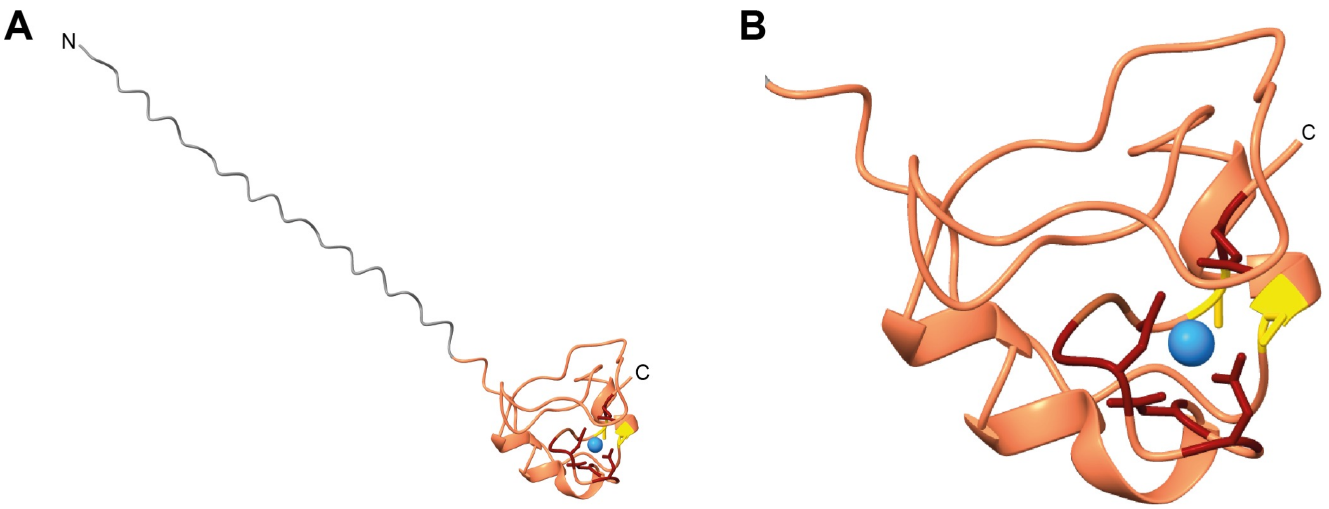

C

MKKYISALLMGTVLIISFSFSTMEVEGAAKVKAVAYKNCTELNKVYKGGVSKDAKTTNKGGKTKYKPFVSPELYKLNEKSDRDNDGIACEK

Figure S2. Structure prediction of the Excalibur Domain Protein using AlphaFold3. The protein's N- and C-termini are labelled. The residues of the Ca^{2+} binding motif DxDxDGxxCE (Rigden et al. 2003) are shown in maroon sticks. The conserved pair of cysteine residues, which likely form a disulfide bond and are proposed to act as a structural constraint (Rigden et al. 2003), are marked as yellow sticks. Using SMART (Schultz et al. 2000), a signal peptide (grey) and the Excalibur domain (salmon) were identified. The protein is predicted to bind one Ca^{2+} ion (blue sphere). Structure predictions were performed by Alphafold3 (Abramson et al. 2024) and visualised using ChimeraX (Meng et al. 2023). The locus tag of the gene encoding this protein in the *S. silvestris* CGN12 genome is AB1K09_06620. **A.** Predicted structure of the full-length protein including the unstructured N-terminal region. **B.** Close-up view of the C-terminal Ca^{2+} binding domain. **C.** Amino acid sequence of the protein. The signal peptide is marked in grey, the conserved cysteine residues in yellow and the Ca^{2+} binding residues in maroon. All residues forming the predicted Excalibur domain are underlined in salmon.

References

Abramson, Josh; Adler, Jonas; Dunger, Jack; Evans, Richard; Green, Tim; Pritzel, Alexander et al. (2024): Accurate structure prediction of biomolecular interactions with AlphaFold 3. In *Nature* 630 (8016), pp. 493–500. DOI: 10.1038/s41586-024-07487-w.

Meng, Elaine C.; Goddard, Thomas D.; Pettersen, Eric F.; Couch, Greg S.; Pearson, Zach J.; Morris, John H.; Ferrin, Thomas E. (2023): UCSF ChimeraX: Tools for structure building and analysis. In *Protein science : a publication of the Protein Society* 32 (11), e4792. DOI: 10.1002/pro.4792.

Rigden, Daniel J.; Jedrzejas, Mark J.; Galperin, Michael Y. (2003): An extracellular calcium-binding domain in bacteria with a distant relationship to EF-hands. In *FEMS microbiology letters* 221 (1), pp. 103–110. DOI: 10.1016/S0378-1097(03)00160-5.

Schultz, J.; Copley, R. R.; Doerks, T.; Ponting, C. P.; Bork, P. (2000): SMART: a web-based tool for the study of genetically mobile domains. In *Nucleic acids research* 28 (1), pp. 231–234. DOI: 10.1093/nar/28.1.231.
