# Supplementary material for: Metabolic Insights Into Microbially Induced Calcite Formation by Bacillaceae for Application in Bio‐Based Construction Materials
Source: Environ Microbiol. 2025 Apr 2;27(4):e70093. doi: 10.1111/1462-2920.70093 (PMC11965779; doi:10.1111/1462-2920.70093)
Supplement: Supplementary file 2 — Table S1. Strains, vectors and plasmids used in this study. Table S2. Oligonucleotides used in this study. Table S3. Overview of whole genome sequencing data of five environmental isolates. Table S4. Genomic comparison of acetate and TCA cycle related genes. Table S5. Genomic comparison of selected calcium homeostasis genes. [file EMI-27-e70093-s003.pdf]

Supplementary tables

Table S1. Strains, vectors and plasmids used in this study

Table S2. Oligonucleotides used in this study

Table S3. Overview of whole genome sequencing data of five environmental isolates

Table S4. Genomic comparison of acetate and TCA cycle related genes

Table S5. Genomic comparison of selected calcium homeostasis genes

***Table S6**. Experimental numerical data for Figures 1-8, available as separate Excel file*

Table S1. Strains, vectors and plasmids used in this study. The used environmental isolates are listed in Table 1.

**Strains**	**Description**	**Source**
A. pseudofirmus	WT	(Nielsen et al. 1995)
S. cohnii	WT	(Spanka and Fritze 1993)
E. coli DH5α	*supE44 ΔlacU169(Φ80lacZΔM15) hsdR17 recA1 endA1 gyrA96 thi-1 relA1*	Laboratory stock
E. coli S-17	*TpR SmR recA thi pro hsdR-M+RP4: 2-Tc:Mu:Km Tn7 λpir*	Laboratory stock
**Vectors**		
pMAD	for generating unmarked deletion/insertion mutants in Gram+ bacteria	(Arnaud et al. 2004)
**Plasmids**		
pG2k-oriT-gfp	contains origin of transfer	David Leak, University of Bath
pMAD-oriT	pMAD containing the origin of transfer from pG2K-oriT-gfp	This study
pMADOriTExcalibur	pMAD-oriT containing the deletion construct for Δ*excalibur*	This study

Table S2. Oligonucleotides used in this study

**Name**	**Description**	**Sequence 5' → 3'** [a]
SG1072	oriT cassette + BamHI fwd	TTTAA**GGATCC**TCTTCTTGATGGAGCGCATG
SG1073	oriT cassette + BamHI rev	TTTAA**GGATCC**CGCACGATATACAGGATTTTG
SG1229	Up-fwd Excalibur NcoI	AATTT**CCATGG**GCAGATGAAACATTC
SG1230	Up-rev Excalibur	***ACAACTCCTA***CATTGGAAAAAACCTCCCAAAC
SG1231	Do-fwd Excalibur	***TTTTCCAATG***TAGGAGTTGTGAAATAGATGAAAATTCTC
SG1232	Do-rev Excalibur NcoI	AATTT**CCATGG**GCTGAGCAATCATTTC
SG1253	qPCR ACS1 (EC 6.2.1.13) fwd	CAGCCCAGTATGGCATCAA
SG1254	qPCR ACS1 (EC 6.2.1.13) rev	AGCCGCTGATCACTTCAATC
SG1257	qPCR Excalibur fwd	TGGAAGTAGAAGGAGCTGCAAA
SG1258	qPCR Excalibur rev	TTCCGCCTTTGTTCGTTGT
SG1259	qPCR SAT fwd	AAATGCTGAGCAACGTATTGG
SG1260	qPCR SAT rev	ATTAATGAAGGCGCATCTGG
SG1261	qPCR ACS2 (EC 6.2.1.1) fwd	CGCAGTTCACAAATCGACAC
SG1262	qPCR ACS2 (EC 6.2.1.1) rev	CATTACGGCCTTCAAACCAG
SG1279	qPCR RpoB' fwd	ACGATAACCGAACGACCAGA
SG1280	qPCR RpoB' rev	GGTCCTGGTAACCGTCCTTT
SG1301	qPCR Calcium ATPase fwd	AGGACGAGCAAGGCTTAACA
SG1302	qPCR Calcium ATPase rev	CAGGTTTAATTCCCGCTTCA
SG1409	Excalibur knockout check F	GGACATTGGCAGCAAAAGG

[a] Restriction sites are in uppercase bold; overhangs for the Gibson Assembly are in uppercase bold and italicised

Table S3. Overview of whole genome sequencing data of five environmental isolates.

	**BA32**	**CGN12**	**PD1_1**	**Psy5**	**UBN2**
Total Mb	5.73 Mb	4.04 Mb	4.56 Mb	5.77 Mb	6.03 Mb
GC content %	39.7%	38.7%	45.7%	39.9%	40.3
Contigs	12	2	2	2	3
Mean Contig Coverage	52.5x	134x	68x	44x	53x
Contig size	5.12 Mb, 474 Kb, 76 Kb, 58 Kb, 2 Kb, 1 Kb, 1 Kb, 0.5 Kb, 0.5 Kb, 0.4 Kb, 0.4 Kb, 0.3 Kb	3.97 Mb, 71 Kb	4.49 Mb, 72 Kb	5.53 Mb, 239 Kb	5.77 Mb, 242 Kb, 10 Kb
Genbank Accession	JBFEAO000000000	JBFEAN000000000	JBFEAM000000000	JBFEAL000000000	JBFEAK000000000

Table S4. Genomic comparison of acetate and TCA cycle related genes, given as locus tag identifiers, in selected species.

Organism	Acetyl-CoA Synthetase (AMP-forming)	Acetyl-CoA Synthetase (ADP-forming)	Acetate kinase	Phosphate acetyltransferase	Succinyl-CoA:acetate CoA transferase
A. pseudofirmus	WEG16239; WEG16252; WEG17604	-	WEG16230	WEG16846	-
S. cohnii	WP_066413159; WP_066420872; WP_094366011	-	WP_066421356	WP_066421944	WP_066418176
BA32	AB1K32_00085; AB1K32_27600	-	AB1K32_00130	AB1K32_21205	-
CGN12	AB1K09_14095; AB1K09_14185	AB1K09_11545	-	-	AB1K09_09305; AB1K09_18240
PD1_1	AB1K19_04130; AB1K19_04215; AB1K19_10845	-	AB1K19_04270	AB1K19_24055	-
Psy5	AB1K18_20090; AB1K18_20145	-	AB1K18_20000	AB1K18_26695	AB1K18_02865
UBN2	AB1K12_20115; AB1K12_201780	-	AB1K12_20030	AB1K12_27310	AB1K12_02960

Table S5. Genomic comparison of selected calcium homeostasis genes, given as locus tag identifiers, in selected species.

Organism	Excalibur domain proteins	F-type Ca^{2+} ATPase	P-type Ca^{2+} ATPase
A. pseudofirmus	-	WEG16779	WEG15677
S. cohnii	-	WP_066418346	WP_066421864
BA32	AB1K32_18280; AB1K32_22100	AB1K32_21550	AB1K32_09520
CGN12	AB1K09_06620; AB1K09_02315	AB1K09_03070	AB1K09_00890
PD1_1	AB1K19_23255	AB1K19_00170	AB1K19_12595
Psy5	-	AB1K18_26420	AB1K18_08120
UBN2	AB1K12_21980	AB1K12_27045	AB1K12_08175

References

Arnaud, Maryvonne; Chastanet, Arnaud; Débarbouillé, Michel (2004): New vector for efficient allelic replacement in naturally nontransformable, low-GC-content, gram-positive bacteria. In *Applied and environmental microbiology* 70 (11), pp. 6887–6891. DOI: 10.1128/AEM.70.11.6887-6891.2004.

Nielsen, P.; Fritze, D.; Priest, F. G. (1995): Phenetic diversity of alkaliphilic Bacillus strains: proposal for nine new species. In *Microbiology* 141 (7), pp. 1745–1761. DOI: 10.1099/13500872-141-7-1745.

Spanka, R.; Fritze, D. (1993): Bacillus cohnii sp. nov., a new, obligately alkaliphilic, oval-spore-forming Bacillus species with ornithine and aspartic acid instead of diaminopimelic acid in the cell wall. In *International journal of systematic bacteriology* 43 (1), pp. 150–156. DOI: 10.1099/00207713-43-1-150.
